# Clinical variables and quantitative CT radiomics for mortality risk stratification after acute exacerbation of idiopathic inflammatory myopathy-associated interstitial lung disease

**DOI:** 10.3389/fimmu.2026.1917500

**Published:** 2026-10-02

**Authors:** Liyu He, Yanrong Chen, Jingping Zhang, Kai Yang, Shuo Yu, Yaqi Yan, Yuhan Bian, Youmin Guo, Chenwang Jin

**Affiliations:** 1 Department of Radiology, The First Affiliated Hospital of Xi’an Jiaotong University, Xi’an, China; 2 Xi’an Key Laboratory of Metabolic Disease Imaging, Xi’an No.3 Hospital, Affiliated Hospital of Northwest University, Xi’an, China; 3 Shaanxi Engineering Research Center of Computational Imaging and Medical Intelligence, Xi’an, China

**Keywords:** acute exacerbation, idiopathic inflammatory myopathy, interstitial lung disease, PaO_2_/FiO_2_, prognostic model, radiomics, risk stratification, SHAP

## Abstract

**Background:**

Acute exacerbation (AE) is the most lethal event of idiopathic inflammatory myopathy-associated interstitial lung disease (IIM-ILD). This exploratory study compared clinical, radiomics, and combined predictor strategies across five survival-learning algorithms for survival risk stratification.

**Methods:**

We retrospectively analyzed 78 patients with AE-IIM-ILD and divided them into a training cohort (n = 54; 20 deaths) and a held-out internal testing cohort (n = 24; 9 deaths), stratified by observed mortality event status. Three predictor strategies were evaluated: a clinical strategy comprising PaO_2_/FiO_2_ ratio, neutrophil percentage (NEU%), and arthritis-related symptoms; a radiomics strategy comprising two selected radiomics features; and a combined strategy comprising PaO_2_/FiO_2_ ratio, arthritis-related symptoms, and a composite radiomics score (Rad-score). Each strategy was evaluated using five survival-learning algorithms. Harrell’s concordance index (C-index) was the primary discrimination metric, with mean time-dependent area under the curve (AUC) used as a secondary measure.

**Results:**

During the 1-year follow-up, 29 deaths were observed. Performance rankings varied across predictor strategies, algorithms, and evaluation metrics. The combined-Random Survival Forest configuration demonstrated the highest training-cohort C-index among the 15 configurations. Its mean time-dependent AUC was 0.923 (95% CI: 0.860–0.970) in the training cohort and 0.915 (95% CI: 0.736–0.986) in the testing cohort, with corresponding C-indices of 0.874 (95% CI: 0.803–0.940) and 0.842 (95% CI: 0.673–0.971), respectively. The model stratified patients into high- and low-risk groups with distinct observed survival trajectories.

**Conclusion:**

In this exploratory cohort, integrating selected clinical variables with a quantitative CT Rad-score using RSF showed promising numerical discrimination and risk-stratification performance. These findings suggest that clinical and quantitative imaging information may provide complementary prognostic information after AE-IIM-ILD.

## 1 Introduction

Interstitial lung disease (ILD) represents the most frequent and critical pulmonary complication of idiopathic inflammatory myopathies (IIM), serving as the primary driver of morbidity and mortality and posing a major challenge for clinical management (1, 2). The prevalence of IIM-ILD ranges from 19.9% to 42.6% (1, 3), with a notably higher incidence in Asian population compared to Europeans (4, 5). While the clinical course varies, approximately 5.7% to 31.9% (6, 7) of patients experience a rapid, fulminant deterioration known as acute exacerbation (AE) (8, 9). Characterized by rapidly worsening dyspnea and new radiological opacities, this catastrophic event carries a short-term mortality rate of 39% to 47% (10–12), making it one of the most lethal complications in the IIM-ILD spectrum.

Previous studies have identified multiple risk factors associated with poor outcomes in AE-ILD, including impaired baseline pulmonary function parameters, severe hypoxemia, and elevated serum biomarkers such as C-reactive protein (CRP), lactate dehydrogenase (LDH), ferritin, Krebs von den Lungen-6 (KL-6), and D-dimer, as well as specific HRCT patterns like usual interstitial pneumonia (UIP) or diffuse alveolar damage (DAD) (6, 9, 10, 13–17). However, the prognostic performance of these variables remains modest, partly due to the substantial heterogeneity across studies. Furthermore, existing risk-stratification frameworks typically rely on isolated pulmonary parameters, omitting extra-pulmonary manifestations that reflect the broader systemic autoimmune activity at AE onset (18, 19). Conventional visual HRCT assessment remains important in ILD evaluation; however, interpretation of individual parenchymal features and ILD patterns is subject to interobserver variability, and visual assessment is not designed to provide the high-dimensional quantitative characterization of image intensity, shape, and texture achievable with computational image analysis (20–22).

Radiomics offers a quantitative approach to characterize imaging phenotypes by extracting high-dimensional features beyond human visual perception, thereby quantifying tissue heterogeneity and texture patterns that may indirectly reflect underlying histopathological changes, including patchy inflammatory infiltration and organizing fibrosis (21, 23). Coupled with machine learning, radiomics becomes a powerful tool for precision diagnosis and prognosis, and has been widely applied in the prognosis prediction of ILD (23–28). For instance, recent multi-center studies by Xu et al. (29) and Li et al. (26) successfully employed radiomics-based ML models to predict mortality in anti-MDA5 positive DM-ILD, reporting higher predictive accuracy compared to clinical variables alone. Despite these advances in stable or sub-acute ILD, radiomics-based prognostic studies specifically targeting AE-IIM-ILD remain scarce.

This study aims to explore the prognostic information provided by clinical variables and quantitative CT radiomics in AE-IIM-ILD to assess the feasibility of integrating these data across multiple survival-learning algorithms.

## 2 Materials and methods

### 2.1 Study design and participants

This retrospective cohort study complied with the Declaration of Helsinki and was approved by the Institutional Review Board of the First Affiliated Hospital of Xi’an Jiaotong University (Approval No. XJTU1AF2024LSYY-268). The requirement for written informed consent was waived due to its observational nature.

We screened 179 consecutive patients with a confirmed diagnosis of IIM-ILD and hospitalized in the Department of Rheumatology and Immunology between 2014 and 2022. The diagnosis of IIM was based on the 2017 EULAR/ACR classification criteria (30) and the 2018 framework proposed by Selva-O’Callaghan et al. (31). Diagnosis of IIM-ILD was independently reassessed and confirmed through multidisciplinary discussion involving experienced pulmonologists, rheumatologists, and thoracic radiologists, integrating clinical presentation, serological profiles, and HRCT findings in accordance with ATS/ERS guidelines (8), while excluding occupational, environmental, or drug-induced ILD.

Inclusion criteria were: i. Age ≥ 18 years; ii. a confirmed diagnosis of IIM-ILD; iii. availability of baseline clinical data and chest HRCT images (within 7 days of admission); iv. availability of follow-up information. AE-IIM-ILD was defined according to previously proposed criteria for rheumatic disease (9), including: *a.* previous or current confirmed diagnosis of ILD; b. worsening or development of dyspnea typically within 1 month; c. new bilateral ground-glass opacities and/or consolidation on chest HRCT superimposed on pre-existing ILD patterns; d. respiratory deterioration not fully explained by cardiac failure, fluid overload, or the use of disease-modifying anti-rheumatic drugs.

Exclusion criteria were: i. absence of chest HRCT, poor image quality, or prior thoracic surgery; ii. coexisting thoracic malignancy; iii. severe organ failure (e.g., heart, liver, or renal failure). Poor image quality was defined as severe respiratory motion or streak artifacts, marked image noise, or inadequate inspiration that substantially impaired visualization of the lung parenchyma or prevented reliable lung segmentation and radiomics feature extraction. Images with minor artifacts that did not interfere with segmentation or feature extraction were retained.

Concurrent pulmonary infection was not considered an exclusion criterion. Patients with suspected or microbiologically confirmed pulmonary infection remained eligible if they otherwise fulfilled the predefined diagnostic criteria for AE-IIM-ILD, because infection may coexist with or act as a trigger for acute exacerbation in rheumatic disease-associated ILD (9). Excluding all infection-associated cases would have reduced the representativeness of this real-world inpatient cohort. Available microbiological findings were retrospectively recorded as described below.

A total of 78 AE-IIM-ILD patients meeting the eligibility criteria were included. Twenty-nine deaths were observed within 1 year after AE onset. The dataset was randomly divided at a ratio of 7:3 into a training cohort (n = 54; 20 deaths) and a testing cohort (n = 24; 9 deaths), stratified solely by observed mortality event status. All feature-selection, preprocessing, and model-development procedures were performed using the training cohort, whereas the testing cohort was used only for internal performance evaluation. The patient-selection process is shown in **Figure 1**. No formal *a priori* sample-size calculation was performed, and all eligible patients identified during the study period were included.

**Figure 1:**
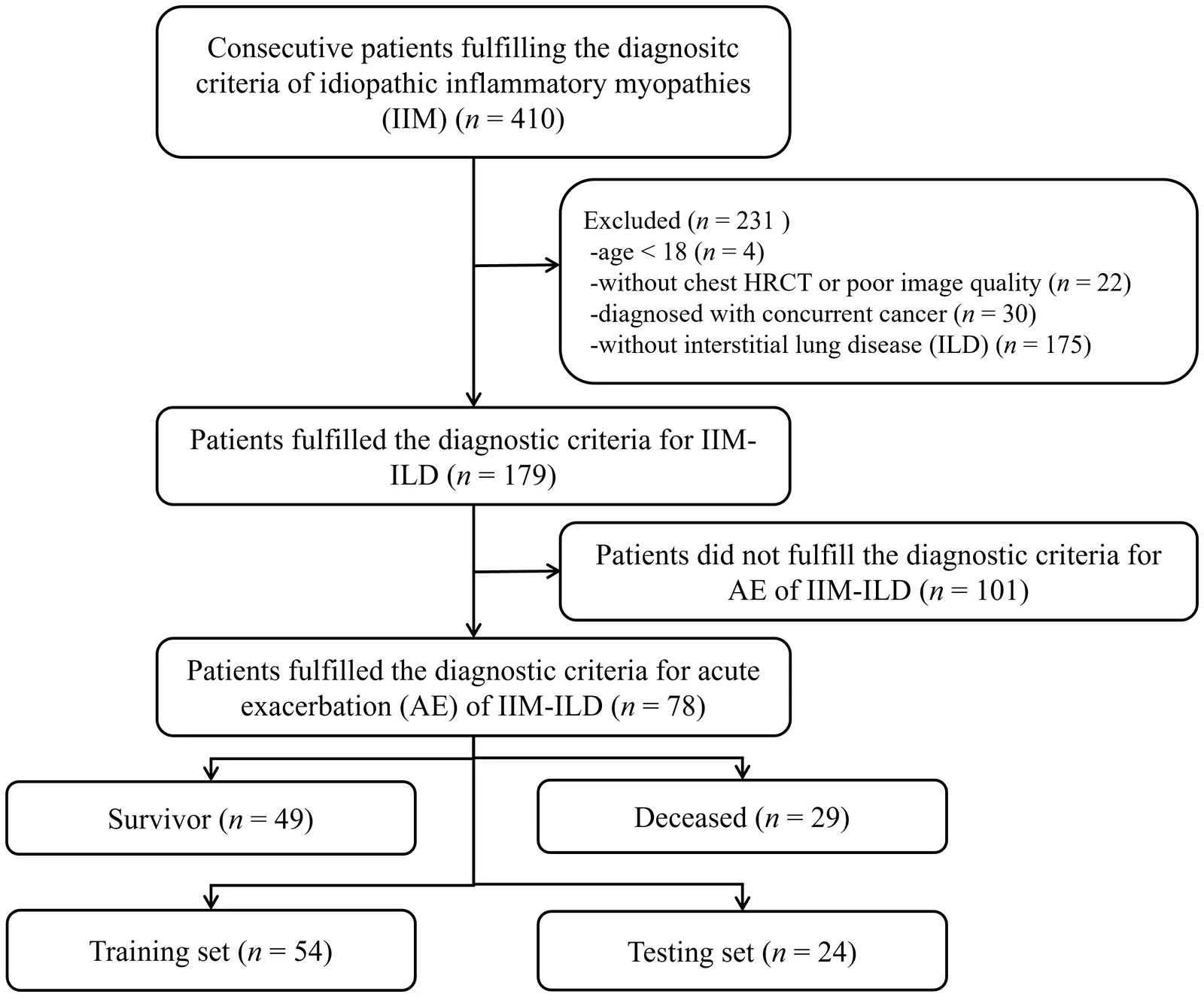
Patient inclusion and exclusion flowchart.

### 2.2 Clinical and laboratory data

Baseline demographic, clinical, laboratory, microbiological, serological, and treatment data were retrospectively collected from medical records. Demographic and medical-history variables included sex, age, disease duration of IIM, smoking history, and comorbidities such as hypertension, diabetes mellitus, coronary artery disease, and other inflammatory or allergic disorders (e.g., allergic rhinitis, sinusitis, asthma, or related conditions). IIM disease duration was obtained from the available clinical history. In contrast, ILD duration before AE admission could not be reliably determined because the date of ILD onset or initial diagnosis was not consistently available, particularly for patients referred after diagnosis or treatment elsewhere. No standardized global myositis disease activity measure was systematically recorded at AE admission. The available data comprised individual clinical manifestations, physical-examination findings, laboratory results, and pulmonary imaging variables.

Information extracted at AE admission included vital signs (temperature, pulse rate, blood pressure, height, and weight) and presenting symptoms/signs, such as fever, cough or sputum production, dyspnea or increased breathlessness, skin manifestations (including Gottron’s papules, heliotrope rash, shawl sign, V-sign, Raynaud phenomenon, skin ulceration, mechanic’s hands), myalgia, muscle weakness, and arthritis-related symptoms (physician-documented joint pain or swelling).

Laboratory parameters obtained within 48 hours of admission included complete blood count, liver function tests, muscle enzyme panel, erythrocyte sedimentation rate (ESR), C-reactive protein (CRP), ferritin, pro–B-type natriuretic peptide (pro-BNP), and arterial oxygenation index (PaO_2_/FiO_2_).

Microbiological investigations, such as sputum and blood cultures, bronchoalveolar lavage fluid (BALF) analyses, respiratory viral PCR tests, and fungal biomarker assays (β-D-glucan, galactomannan), were also recorded.

Serological data regarding myositis-specific antibodies (MSAs) and myositis-associated antibodies (MAAs) from the first admission were collected. MSA/MAA test results were available for 55 patients. Testing was performed using a semiquantitative immunoblotting method, with individual antibody results reported in arbitrary units and interpreted according to laboratory-defined thresholds. None of the patients underwent immunoprecipitation-based antibody testing.

Therapeutic interventions during the AE event were documented, including corticosteroid regimens, use of disease-modifying antirheumatic drugs (DMARDs), oxygen therapy (supplemental oxygen or no oxygen), and mechanical ventilation requirements.

### 2.3 Follow-up and outcomes

Follow-up information was obtained through hospital records, outpatient visits, or telephone interviews. Follow-up time was calculated from the onset of AE until death, the last confirmed contact, or 365 days, whichever occurred first. The primary outcome was 1-year cumulative survival following the AE episode. Cumulative survival probabilities at 30-day, 90-day, and 180-day were estimated using the Kaplan-Meier method.

During the 1-year follow-up period, 29 deaths were observed, 47 patients were confirmed to be alive at 1 year, and 2 patients were lost to follow-up and right-censored at 8 and 10 months, respectively.

### 2.4 HRCT acquisition and visual evaluation

Baseline HRCT images obtained within 7 days after admission for AE were included in the analysis. All chest HRCT examinations were performed without intravenous contrast enhancement using ≥ 64-slice CT scanners, with patients in the supine position during an end-inspiratory breath-hold. The acquisition parameters were as follows: tube voltage 120 kV, automatic tube current modulation, and a matrix size of 512 × 512 or 1024 × 1024. The scanning range covered the entire lungs from the apex to the lung bases.

Images were reconstructed using a high-spatial-frequency bone-kernel reconstruction with a slice thickness of 1.0 mm and a reconstruction interval of 1.0 mm. The bone-kernel series represented the routinely acquired thin-section HRCT reconstructions used for clinical evaluation of fine pulmonary parenchymal structures and consistently available for the included examinations. Images were transferred to the workstation in DICOM format, anonymized, and reviewed in standard lung (window width/level = 1500/−600 HU) and mediastinal windows (400/50 HU).

Visual ILD patterns at AE onset were independently evaluated by two chest radiologists with more than 10 years of experience, with disagreements resolved by consensus. Patterns were evaluated with reference to the morphological definitions described in the 2013 ATS/ERS multidisciplinary classification statement (8) and previous IIM-ILD literature (2, 32). Because different morphological patterns may coexist in IIM-ILD, overlap patterns were recorded when features of more than one pattern were present on the same examination. For analysis, the observed patterns in this cohort were classified into five mutually exclusive categories: nonspecific interstitial pneumonia (NSIP), organizing pneumonia (OP), overlapping NSIP+OP, DAD, and overlapping UIP+OP.

### 2.5 Image segmentation and radiomics feature extraction

The original HRCT data were semi-automatically segmented in 3D Slicer (RRID: SCR_005619) (https://www.slicer.org/). A semi-automated approach with manual correction was selected because the complex and heterogeneous pulmonary abnormalities present during AE could obscure anatomical boundaries and lobar fissures in AI models. Briefly, a radiologist with more than 5 years of experience contoured the anatomical boundaries of each lung lobe across sparse axial CT slices (spaced 1~3 slices) using the Paint tool. The Fill between slices module was then executed to perform automated 3D morphological interpolation, filling unannotated gaps between slices. Surface artifacts were polished using the Smoothing (Joint Smoothing) module, and boundary errors were manually refined using the Scissors and Erase tools. Five individual lobar volumes of interest (VOIs), the right upper, right middle, right lower, left upper, and left lower lobes were segmented separately and subsequently merged via logical operations to construct left-lung, right-lung, and whole-lung VOIs (yielding 8 VOIs per subject). All final masks were audited and approved by a senior radiologist with >10 years of experience.

After segmentation, images were resampled to an isotropic voxel size of 1×1×1 mm using linear interpolation. Intensity discretization was performed with a fixed bin width of 25 to standardize intensity discretization. Wavelet decomposition was applied using PyWavelets (RRID: SCR_024421) to mitigate scanner-related noise and generate three sets of reconstructed images: high-, medium-, and low-frequency components (representing edges/detail, structural detail with some noise, and overall background structure, respectively).

Radiomics features were extracted using PyRadiomics (version 3.0.1, RRID: SCR_026019) from each VOI, including 18 first-order features, 8 shape features, and 75 texture features. Texture features comprised 24 gray-level co-occurrence matrix features (GLCM), 14 gray-level dependence matrix features (GLDM), 16 gray-level run-length matrix features (GLRLM), 16 gray-level size-zone matrix features (GLSZM), and 5 neighboring gray-tone difference matrix features (NGTDM). The same feature sets were extracted from each of the three wavelet-filtered image sets. In total, 3232 radiomics features were obtained for each subject.

### 2.6 Feature selection and predictor-strategy definition

To minimize the risk of overfitting and information leakage, all feature preprocessing and selection procedures were performed exclusively in the training cohort. The testing cohort was not involved in feature selection or predictor-strategy definition and was reserved for held-out internal performance evaluation.

Clinical feature selection: Candidate clinical, laboratory, and visual HRCT parameters were initially examined in the training cohort. Variables describing treatments administered after AE onset were summarized for descriptive purposes but were not eligible for inclusion in the final baseline predictor sets. Among the eligible baseline clinical, laboratory, and visual HRCT variables, nine showed significant differences between patients with and without an observed death (*P* < 0.05). One of these variables, “MSA/MAA testing unavailable,” indicated the absence of serological testing results rather than a biological patient characteristic and was therefore excluded from subsequent prognostic modeling. The remaining eight variables were evaluated using univariable Cox proportional hazards regression. Five variables showed significant univariable associations with mortality: PaO_2_/FiO_2_ ratio, NEU%, arthritis-related symptoms, lymphocyte percentage, and C-reactive protein. Five-fold cross-validated feature-importance ranking using Cox proportional hazards and Random Survival Forest algorithms subsequently identified PaO_2_/FiO_2_ ratio, NEU%, and arthritis-related symptoms as the three highest-ranked clinical features. These three variables constituted the clinical predictor strategy. The variables evaluated and retained at each stage of the clinical feature-selection process are provided in **Supplementary Table S2**.

Radiomics feature selection and Rad-score construction: The 3232 radiomics features extracted from 8 VOIs per subject were sequentially reduced using: (1) low-variance filtering, with features showing variance <0.01 removed; (2) between-group statistical screening, with features showing *P* < 0.05 retained; (3) hierarchical clustering based on the Pearson correlation matrix, with redundant features showing |r| > 0.8 removed; (4) univariate Cox regression screening with *P* < 0.05; and (5) 5-fold cross-validated LASSO-Cox L1 penalization to shrink small coefficients. The top-ranked dominant radiomics features identified by cross-validated importance scoring were selected, and a linear Radiomics Score (Rad-score) was calculated for each patient weighted by their respective LASSO regression coefficients in the training cohort. Details are presented in **Supplementary Figure S1** and **Supplementary Table S4**.

Predictor-strategy definition: Three predictor strategies were defined before algorithm benchmarking. The clinical strategy comprised PaO_2_/FiO_2_ ratio, arthritis-related symptoms, and NEU%. The radiomics strategy comprised the two selected radiomics features entered separately. For the combined strategy, the Rad-score was used as a single composite radiomics variable and was initially assessed together with the three clinical variables. Multicollinearity assessment showed VIF values of 5.38 for NEU% and 5.24 for PaO_2_/FiO_2_ ratio (**Supplementary Table S3**). NEU% was excluded from the combined strategy because of its collinearity with PaO_2_/FiO_2_ ratio and its nonsignificant association in the multivariable clinical Cox analysis (*P* = 0.10), whereas PaO_2_/FiO_2_ ratio was retained because of its clinical relevance and stronger association with survival. The variance inflation factors, tolerance values, and corresponding decisions for the combined predictor are provided in **Supplementary Table S3**. The final combined predictor therefore comprised PaO_2_/FiO_2_ ratio, arthritis-related symptoms, and the Rad-score.

### 2.7 Model development and evaluation

Using the three predefined predictor strategies, five survival-learning algorithms were evaluated separately: Cox proportional hazards regression (CoxPH), Fast Kernel Survival Support Vector Machine (survival SVM), Gradient Boosting Survival Analysis (GBSA), Random Survival Forest (RSF), and Extra Survival Trees (EST). The clinical strategy comprised PaO_2_/FiO_2_ ratio, NEU%, and arthritis-related symptoms; the radiomics strategy comprised the two selected radiomics features entered separately; and the combined strategy comprised PaO_2_/FiO_2_ ratio, arthritis-related symptoms, and the Rad-score. This design yielded 15 model configurations comprising all combinations of the three predictor strategies and five algorithms.

All model fitting, preprocessing, and hyperparameter optimization were restricted to the training cohort. Where feature scaling was required, preprocessing parameters were estimated using the training cohort and applied unchanged to the testing cohort. Hyperparameters for the four machine-learning algorithms were optimized using grid search with five-fold cross-validation in the training cohort, with the concordance index as the optimization metric. CoxPH was fitted without algorithm-specific hyperparameter tuning. The testing cohort was not used during model fitting or hyperparameter optimization and was used only for held-out internal performance evaluation.

Harrell’s concordance index (C-index) was used as the primary measure of discrimination and was calculated separately in the training and testing cohorts. Confidence intervals were estimated using bootstrap resampling. Cumulative/dynamic time-dependent AUC curves were additionally generated over the evaluable follow-up range of each cohort using inverse-probability-of-censoring weighting. Because follow-up was administratively truncated at 365 days, the AUC at the exact 365-day boundary was not estimated. The mean time-dependent AUCs summarized discrimination over the displayed evaluable time interval and were treated as secondary descriptive measures.

For exploratory risk stratification, a risk-score cutoff was derived in the training cohort by maximizing the log-rank statistic while requiring at least five patients in each risk group. The training-derived cutoff was applied unchanged to the testing cohort, and survival differences between the resulting risk groups were assessed using Kaplan–Meier curves and the log-rank test. Shapley Additive Explanations (SHAP) were used to visualize feature contributions to predictions from the Combined-RSF configuration; SHAP values were interpreted as descriptions of model behavior rather than as evidence of causal or biological mechanisms.

### 2.8 Statistical analysis

All statistical analyses were conducted using Python 3.8 (RRID: SCR_008394) with the following packages: SimpleITK (RRID: SCR_024693), PyTorch (RRID: SCR_018536), scikit-learn (RRID: SCR_002577), scikit-feature (RRID: SCR_016141), SciPy (RRID: SCR_008058), and pyradiomics (RRID: SCR_026019).

Continuous variables were summarized as mean ± standard deviation (SD) or median with interquartile range (IQR), depending on data distribution. Normality was assessed using the Shapiro–Wilk test. Between-group comparisons were performed using the Student’s t-test for normally distributed variables or the Mann–Whitney U test for non-normally distributed variables.

Categorical variables were reported as counts and percentages, and compared using the Pearson chi-square test or Fisher’s exact test, as appropriate.

Survival outcomes were analyzed using the Kaplan–Meier method with the log-rank test for group comparisons. A two-tailed P < 0.05 was considered statistically significant.

## 3 Results

### 3.1 Comparison of clinical characteristics

A total of 78 patients with AE-IIM-ILD were included, comprising 21 males (26.9%) and 57 females (73.1%), with a mean age of 52.4 ± 10.7 years. The cohort consisted of 53 patients with dermatomyositis (DM), 8 with anti-synthetase syndrome (ASyS), 10 with polymyositis (PM), and 7 with overlap myositis (OM).

During the 12-month follow-up period, 29 deaths were observed. Forty-seven patients were confirmed to be alive at 1 year, whereas two patients were lost to follow-up and right-censored at 8 and 10 months, respectively. For descriptive baseline comparisons, patients were categorized according to observed event status as those with and without an observed death; censored observations were retained in the latter group. Kaplan–Meier analysis (**Figure 2**) showed that the 30-day, 90-day, 180-day, and 1-year cumulative survival rates were 80.8% (95% CI: 72.5–90.0%), 75.6% (95% CI: 66.7–85.8%), 69.2% (95% CI: 59.7–80.3%), and 62.8% (95% CI: 52.9–74.5%), respectively.

**Figure 2:**
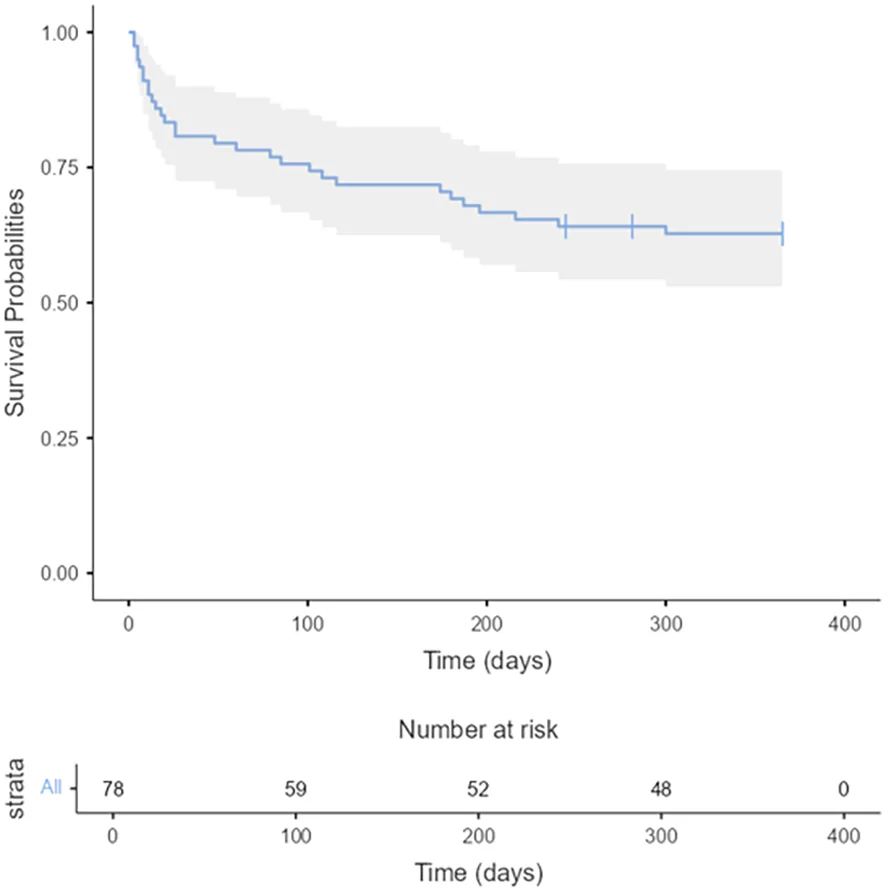
AE-IIM-ILD patient Kaplan-Meier analysis. AE, acute exacerbation; IIM, idiopathic inflammatory myopathies; ILD, interstitial lung disease.

Compared with patients without an observed death, patients with an observed death exhibited a significantly lower PaO_2_/FiO_2_ ratio (175.2 vs. 260.1 mmHg; p<0.001), a higher prevalence of arthritis-related symptoms (44.8% vs. 16.3%; p=0.006), and a higher Rad-score (0.38 vs. -0.08; p<0.001) at AE onset.

The 78 patients were randomly divided into a training set (n = 54; 34 patients without an observed death) and a testing set (n = 24; 15 patients without an observed death). There was no significant difference in 1-year mortality between the training and testing sets (37.1% vs. 37.5%). The comparison of selected clinical, laboratory, and radiological characteristics is summarized in **Table 1** (full characteristics available in **Supplementary Table S1**).

**Table 1:** Clinical, laboratory, and radiologic characteristics of the study population in training and testing cohorts.

Characteristics§	Training cohort (*n* = 54)		*P* value	Testing cohort (*n* = 24)		*P* value
	No observed death (*n* = 34)	Death (*n* = 20)		No observed death (*n* = 15)	Death (*n* = 9)	
Demographics						
Age, years	53.1(12.4)	53.6(8.8)	0.856	48.9(9.9)	53.3(9.2)	0.291
Female, n (%)	23(67.6)	15(75)	0.568	13(86.7)	6(66.7)	0.326#
BMI, kg/m^2^	22(2.6)	20.5(4.1)	0.209	22.9(2.8)	19.9(2.7)	**0.017**
IIM subtypes, n (%)						
DM	25(73.5)	12(60)	0.301	9(60)	7(77.8)	0.657#
Clinical Signs, n (%)						
Dyspnea	13(38.2)	9(45)	0.625	11(73.3)	4(44.4)	0.212#
Arthralgia/Joint swelling	6(17.6)	10(50)	**0.012**	2(13.3)	3(33.3)	0.326#
Pulse, bpm	94(17.2)	100(17.8)	0.189	86(31.5)	102(34)	**0.034**
Laboratory Findings						
PaO_2_/FiO_2_, mmHg	263(91.9)	183(85.4)	**0.003**	254(83.5)	159(86.9)	**0.014**
Hemoglobin, g/L	128(20)	116(18.3)	0.014*	126(12.8)	120(20.9)	0.422
Lymphocyte%	12.9(15.7)	7.7(6.5)	0.004*	10.2(6.9)	9.5(5.3)	0.411*
Neutrophil, 10^9^/L	4.6(3.6)	6.2(3.6)	0.025*	6.1(2.6)	7.4(4.0)	0.482*
Neutrophil %	77.8(19.5)	87.8(7.1)	**<0.001**	80.1(8.5)	83(6.4)	0.395
PCT, ng/ml	0.05(0.01)	0.11(0.34)	0.025*	0.05(0.12)	0.08(0.13)	0.352*
CRP, mg/L	10(43.7)	48.1(66.3)	0.038*	10(28.7)	12.1(41)	0.261*
Treatment & Comorbidities						
IVIG	5(14.7)	8(40)	0.05#	0(0)	3(33.3)	**0.042#**
Mechanical Ventilation	3(8.8)	4(20)	0.403#	1(6.7)	5(55.6)	**0.015#**
Comorbidities (Any)	20(58.8)	12(60)	0.932	4(26.7)	7(77.8)	**0.033#**

§Data are presented as mean (SD) or median [IQR] for continuous variables as appropriate, and number (%) for categorical variables. * Mann-Whitney U test; # Fisher’s exact test. Note: Selected variables are shown. Complete demographic and clinical data are provided in **Supplementary Table S1**. Bold indicates P < 0.05.

Significant differences were observed between patients with and without an observed death across both cohorts. Notably, the PaO_2_/FiO_2_ was significantly lower in the deceased group compared to patients without an observed death in both the training set (P = 0.003) and the testing set (P = 0.014). Regarding treatment, patients in the deceased group received intravenous immunoglobulin (IVIG) more frequently; this difference was of borderline significance in the training set (P = 0.05) and statistically significant in the testing set (P = 0.042).

In the training set, the deceased group exhibited a significantly higher prevalence of arthritis-related symptoms (arthralgia/joint swelling) (P = 0.012). Furthermore, inflammatory markers including neutrophil percentage (NEU%), C-reactive protein (CRP), and procalcitonin (PCT) were significantly elevated in patients with an observed death (P < 0.05), while hemoglobin levels and lymphocyte percentage were significantly lower compared to patients without an observed death (P < 0.05). Although NEU% showed significant between-group differences and was retained in the clinical predictor, it was excluded from the combined strategy because of its collinearity with PaO_2_/FiO_2_ and its attenuated association in the multivariable clinical CoxPH analysis.

In the testing set, patients with an observed death presented with significantly higher pulse rates and lower BMI compared to patients without an observed death (P < 0.05). They also had a higher burden of comorbidities and a significantly higher requirement for mechanical ventilation (P = 0.015).

### 3.2 Feature selection

Clinical predictor selection: Among the 68 clinical, laboratory, treatment-related, and visual HRCT variables reported in **Supplementary Table S1**, nine showed significant between-group differences in the training cohort. The variable indicating unavailable MSA/MAA testing (Miss) was excluded because it represented data availability rather than a biological or clinical characteristic. Among the remaining variables, univariable Cox regression identified five variables associated with survival: PaO_2_/FiO_2_ ratio, NEU%, arthritis-related symptoms, lymphocyte percentage, and C-reactive protein. Cross-validated feature-importance ranking using CoxPH and Random Survival Forest algorithms subsequently identified PaO_2_/FiO_2_ ratio, arthritis-related symptoms, and NEU% as the three leading clinical features (**Figures 3a, b**). These three variables constituted the clinical predictor. The complete stepwise clinical feature-selection process is summarized in **Supplementary Table S2**. Additional univariable CoxPH analyses of the standalone prognostic performance of the three selected clinical variables are presented in **Supplementary Table S5**.

**Figure 3:**
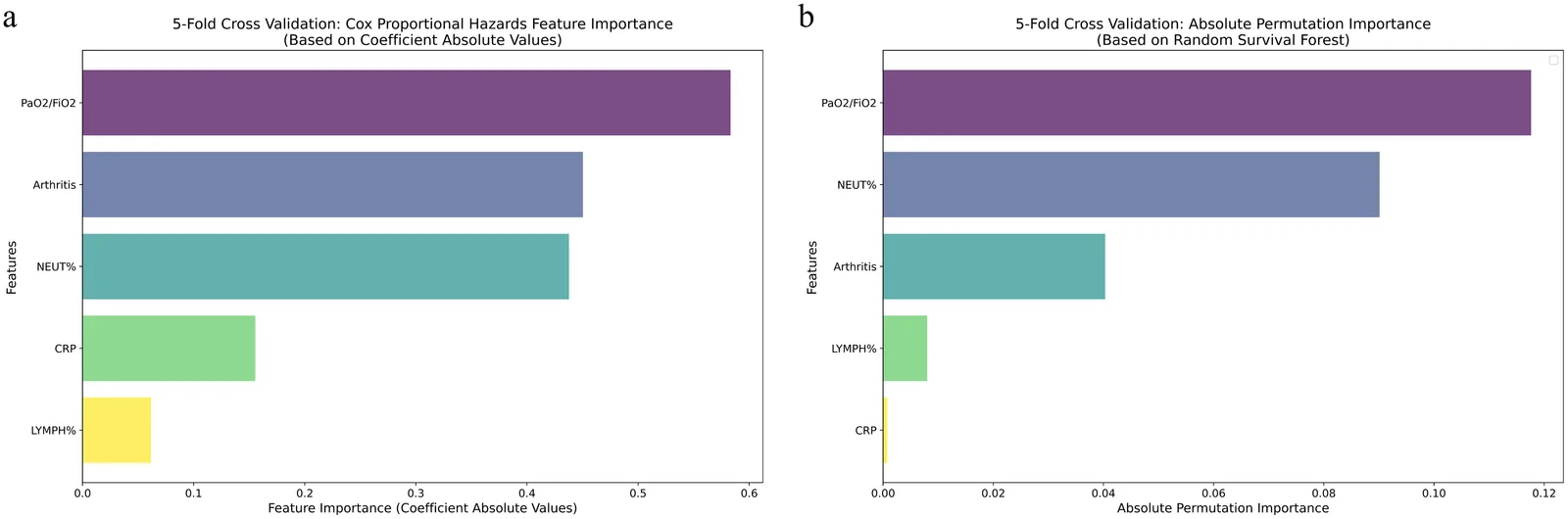
Selection of key clinical predictors. **(a)** Feature importance ranking derived from 5-fold cross-validated Cox proportional hazards regression. **(b)** Feature importance ranking derived from Random Survival Forests (RSF).

Baseline visual ILD-pattern distribution was not significantly different between the outcome groups in the training cohort (overall Fisher’s exact P = 0.603) and was therefore not advanced through the subsequent clinical feature-selection steps.

Radiomics feature selection and Rad-score construction: The multi-tier selection pipeline reduced the 3232 initial radiomics features to 2223 after low-variance filtering, 479 after between-group statistical screening, 50 after hierarchical correlation clustering, 39 after univariable Cox regression, and 6 after cross-validated LASSO-Cox penalization (**Supplementary Table S4**; **Supplementary Figure S1**). Cross-validated feature-importance ranking ultimately identified two radiomics features: MF_RtLower_original_glszm_ZoneEntropy and RAW_RtUpper_original_shape_Maximum2DDiameterSlice. These two features were used directly as predictors in the radiomics strategy. A Rad-score was also calculated as their linear combination weighted by the corresponding LASSO-Cox coefficients for use in the combined strategy.

Accordingly, three predictor strategies were defined before algorithm comparison: (1) the clinical strategy, comprising PaO_2_/FiO_2_ ratio, NEU%, and arthritis-related symptoms; (2) the radiomics strategy, comprising the two selected radiomics features; and (3) the combined strategy, comprising PaO_2_/FiO_2_ ratio, arthritis-related symptoms, and the Rad-score. When constructing the combined predictor set, multicollinearity was reassessed after integrating the Rad-score (**Supplementary Table S3**). NEU% and PaO_2_/FiO_2_ ratio showed variance inflation factors exceeding 5, and NEU% showed an attenuated association in the multivariable Cox analysis (P = 0.10). NEU% was therefore omitted from the combined strategy to reduce redundancy and limit model complexity.

### 3.3 Comparative performance across predictor strategies and algorithms

Five survival-learning algorithms were evaluated under each of the clinical, radiomics, and combined predictor strategies, resulting in 15 model configurations. Their comparative performance is presented in **Figure 4** and **Table 2**. Enlarged strategy-specific results are provided in **Supplementary Figures S2**-**S4**, and complete numerical estimates for all 15 configurations are reported in **Supplementary Table S6**.

**Figure 4:**
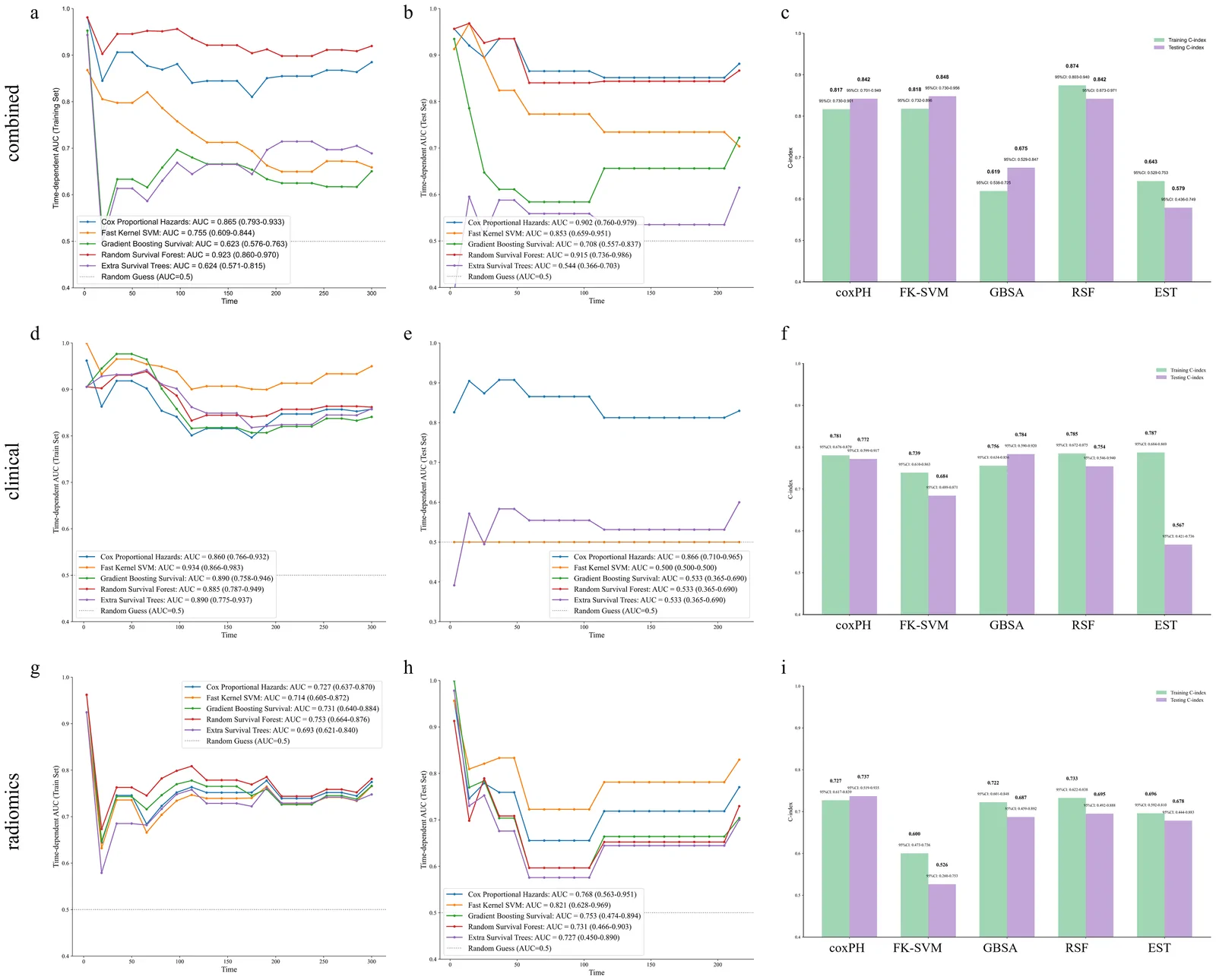
Comparative discrimination of the 15 survival-model configurations under the three predictor strategies. Rows correspond to the combined **(a–c)**, clinical **(d–f)**, and radiomics **(g–i)** predictor strategies, respectively. The first and second columns show time-dependent AUC curves in the training and held-out testing cohorts, respectively, and the third column shows the corresponding C-indices with 95% confidence intervals. Values reported in the curve legends represent the mean time-dependent AUCs with 95% confidence intervals over the displayed evaluable follow-up intervals. The horizontal dotted line represents random discrimination (AUC = 0.5). AUC, area under the curve; C-index, concordance index; CoxPH, Cox proportional hazards regression; FK-SVM, Fast Kernel Survival Support Vector Machine; GBSA, Gradient Boosting Survival Analysis; RSF, Random Survival Forest; EST, Extra Survival Trees.

**Table 2:** Predictive performance of the highlighted model configurations.

Model	Cohort	Mean time-dependent AUC (95% CI)	C-Index (95% CI)
Clinical-CoxPH	Training	0.860 (0.766–0.932)	0.781 (0.676-0.879)
	Testing	0.866 (0.710–0.965)	0.772 (0.599-0.917)
Radiomics-CoxPH	Training	0.727 (0.637-0.870)	0.727 (0.617-0.839)
	Testing	0.768 (0.563-0.951)	0.737 (0.519-0.935)
Combined-RSF	Training	**0.923 (0.860-0.970)**	**0.874 (0.803-0.940)**
	Testing	**0.915 (0.736-0.986)**	**0.842 (0.673-0.971)**

Bold values indicate the highest performance metrics achieved among the evaluated configurations.

Within the clinical predictor, the CoxPH configuration showed similar C-indices between the two cohorts. Its mean time-dependent AUC was 0.860 (95% CI: 0.766–0.932) in the training cohort and 0.866 (95% CI: 0.710–0.965) in the testing cohort, with corresponding C-indices of 0.781 (95% CI: 0.676–0.879) and 0.772 (95% CI: 0.599–0.917), respectively. The performance of the other clinical configurations varied more substantially between the training and testing cohorts.

For the radiomics predictor, performance rankings differed between the C-index and mean time-dependent AUC. The CoxPH configuration yielded C-indices of 0.727 (95% CI: 0.617–0.839) and 0.737 (95% CI: 0.519–0.935) in the training and testing cohorts, respectively, with corresponding mean time-dependent AUCs of 0.727 (95% CI: 0.637–0.870) and 0.768 (95% CI: 0.563–0.951). Although the Fast Kernel Survival SVM achieved a higher mean time-dependent AUC in the testing cohort, its testing C-index was substantially lower, indicating metric-dependent variability rather than uniformly better performance. Overall, the CoxPH configuration provided the most balanced concordance-based performance for the radiomics predictor.

Among the 15 evaluated configurations, the combined-RSF achieved the highest training-cohort C-index (**Supplementary Table S6**). This configuration incorporated the PaO_2_/FiO_2_ ratio, arthritis-related symptoms, and the Rad-score. Its mean time-dependent AUC was 0.923 (95% CI: 0.860–0.970) in the training cohort and 0.915 (95% CI: 0.736–0.986) in the testing cohort. The corresponding C-indices were 0.874 (95% CI: 0.803–0.940) and 0.842 (95% CI: 0.673–0.971), respectively. On this basis, the combined-RSF was carried forward for exploratory risk stratification and model-interpretation analyses. Detailed coefficients, hazard ratios, 95% confidence intervals, z statistics, and P values for the clinical, radiomics, and auxiliary combined CoxPH configurations are provided in **Supplementary Table S7**.

### 3.4 Model interpretability and risk stratification

The Combined-RSF configuration was further evaluated for exploratory risk stratification, and model interpretation. To verify the stratification capability, patients were classified into High-Risk and Low-Risk groups using an optimal risk score cutoff of 7.10 that was determined by maximizing the log-rank statistic in the training set. Kaplan-Meier analysis (**Figure 5**) confirmed significantly distinct outcomes. The High-Risk group had a significantly lower cumulative survival rate in both the training set (Hazard Ratio [HR] = 11.72, P < 0.001) and the held-out testing set (HR = 6.67, P < 0.001).

**Figure 5:**
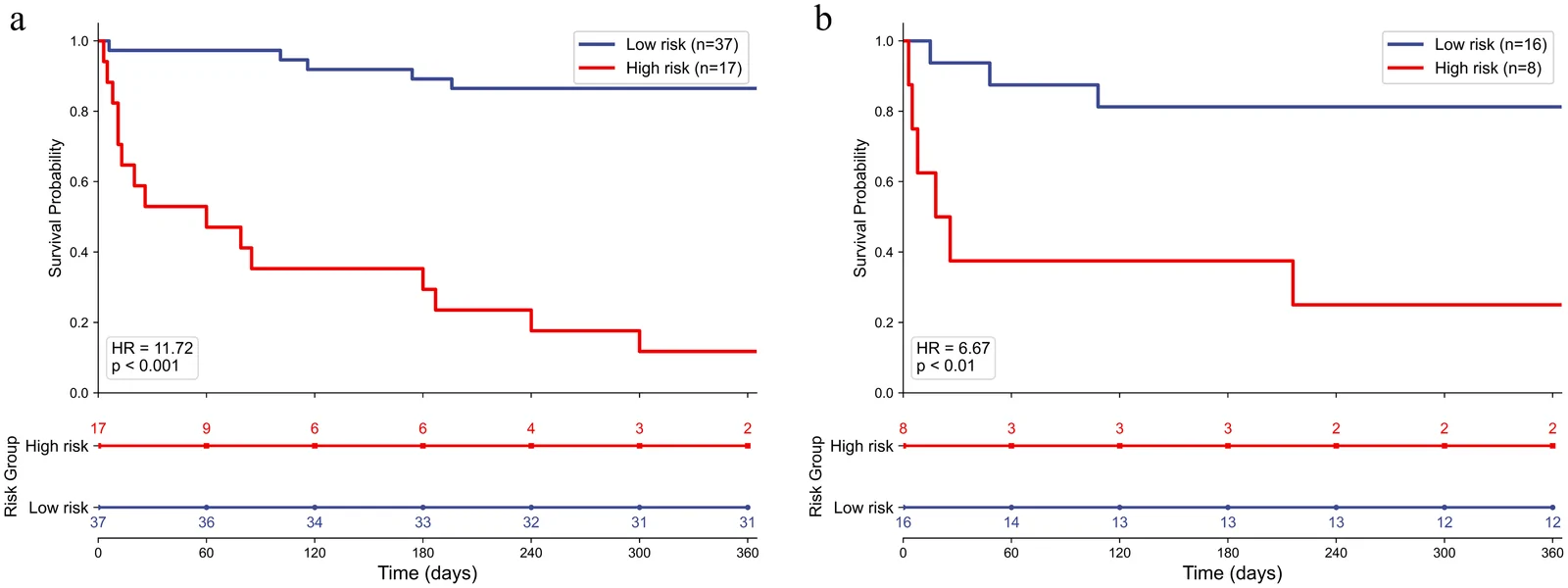
Kaplan-Meier curves stratified by the Combined-RSF model’s risk score cutoff (7.10) in the **(a)** Training and **(b)** Testing sets.

SHAP analysis was used to examine the contributions of the three predictors to the Combined-RSF predictions. The summary plot (**Figure 6a**) showed that PaO_2_/FiO_2_ made the largest contribution to the Combined-RSF predictions, with lower values generally associated with higher mortality risk. The presence of arthritis-related symptoms and a higher Rad-score also contributed toward higher predicted risk. Individual force plots illustrate the decision process for representative patients. For a Low-Risk survivor in the test set (**Figure 6c**), the prediction reflected contributions from a relatively higher PaO_2_/FiO_2_ (161.2 mmHg), a low Rad-score (-0.122), and absence of arthritis. In contrast, for a High-Risk non-survivor (**Figure 6d**), the mortality risk was driven by a low PaO_2_/FiO_2_ ratio (PaO_2_/FiO_2_ 55.9 mmHg), arthritis-related symptoms, and a higher Rad-score (Rad-score 1.573).

**Figure 6:**
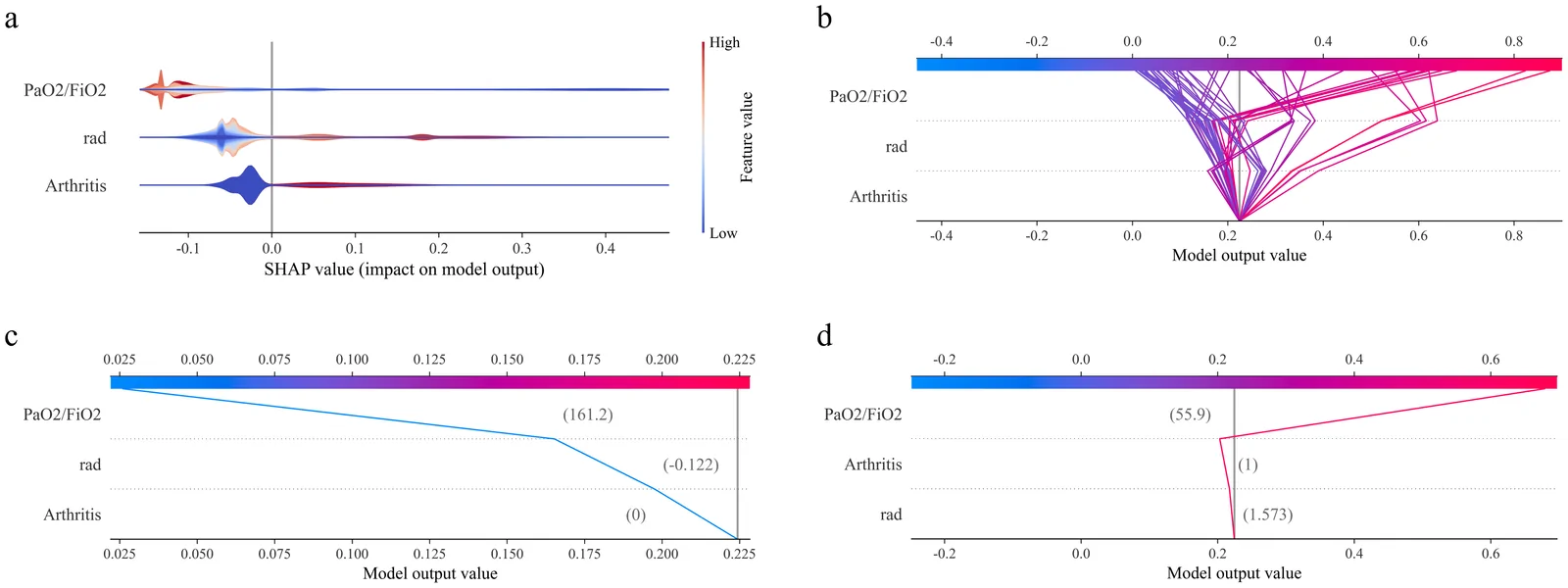
Model interpretability (SHAP). **(a)** Beeswarm summary plot. **(b)** Decision plot. **(c)** Force plot (survivor case). **(d)** Force plot (non-survivor case).

## 4 Discussion

In this exploratory, single-center retrospective study, we developed and internally evaluated a multi-modal prognostic model in 78 patients with AE-IIM-ILD by integrating clinical variables and quantitative radiomics signatures. Three variables, PaO_2_/FiO_2_, arthritis-related symptoms and Rad-score, constituted the predictor set of the final combined-RSF model. Complementary CoxPH analyses showed that a higher PaO_2_/FiO_2_ ratio during AE was associated with a lower mortality hazard, whereas the presence of arthritis-related symptoms and a higher Rad-score were associated with poor outcomes. The combined-RSF model showed favorable discrimination across both the training (AUC 0.923, 95% CI: 0.860–0.970) and the held-out internal testing subset (AUC 0.915, 95% CI: 0.736–0.986). However, given the limited sample size, small number of deaths, wide confidence intervals, and absence of external validation, these performance estimates should be regarded as preliminary.

The oxygenation index (PaO_2_/FiO_2_) is a central physiological marker widely used in respiratory and critical care practice to quantify the impairment of pulmonary gas exchange. It is essential for the diagnosis and severity grading of acute respiratory distress syndrome (ARDS) and is routinely applied to assess disease severity and predict outcomes (33). Previous studies of AE-ILD (34) and AE-CTD-ILD (6) have reported an association between lower PaO_2_/FiO_2_ values during AE and higher mortality risk. Consistent with these findings, a higher PaO_2_/FiO_2_ ratio was associated with a lower mortality hazard in both the clinical CoxPH model and the auxiliary combined CoxPH analysis in the present cohort. Thus, PaO_2_/FiO_2_ represents a readily available measure of acute hypoxemic severity in AE-IIM-ILD. However, the present study did not evaluate whether model-guided respiratory management improves patient outcomes.

Arthritis-related symptoms, defined as physician-documented arthralgia and/or joint swelling at AE admission, were associated with mortality in AE-IIM-ILD. Arthritis-related symptoms were associated with a higher mortality hazard in both the clinical CoxPH model and the auxiliary combined CoxPH analysis. Previous studies suggest that musculoskeletal manifestations may accompany systemic disease activity in IIM (1, 35), and Liang et al. demonstrated that high disease activity scores and low DL_CO_% were significantly correlated with short-term adverse outcomes in AE-IIM-ILD (7). Arthritis-related symptoms at AE onset may be associated with broader systemic disease activity. However, formal disease-activity scores and longitudinal inflammatory assessments were not available in the present cohort. Therefore, whether arthritis-related symptoms reflect broader systemic autoimmune activity or contribute biologically to pulmonary deterioration remains uncertain and should be regarded as a hypothesis rather than a mechanism demonstrated by this study.

Radiomics extracts high-dimensional quantitative features from HRCT images to characterize image-intensity distributions, morphology, and tissue heterogeneity beyond conventional visual interpretation (21) and has shown potential prognostic value in various ILD settings (28, 36–38). For instance, He et al. (25) demonstrated that radiomics signatures are reliable predictors of mortality in anti-MDA5-positive DM-ILD, while studies in broader CTD-ILD cohorts have evaluated the prognostic contribution of radiomics alongside clinical risk factors (23, 24). Recently, a multi-center study by Lu et al. developed a combined clinical-radiomics nomogram to predict poor prognosis in a general IIM-ILD cohort, achieving a validation AUC of 0.88 (19). In contrast to studies focusing on relatively stable or chronic ILD, the present study focused specifically on survival after AE-IIM-ILD. We evaluated five survival-learning algorithms separately under clinical, radiomics, and combined predictor strategies, yielding 15 model configurations. Performance rankings varied according to predictor, algorithm, and evaluation metric. CoxPH showed comparatively balanced performance across the training and testing cohorts for the clinical and radiomics strategies, whereas the combined strategy performed best using RSF. These findings suggest that clinical and quantitative imaging information may provide complementary prognostic information, although their incremental value requires confirmation in larger externally validated cohorts.

Conventional visual HRCT assessment remains clinically important in AE-IIM-ILD. In the present cohort, visual ILD pattern was evaluated as an initial candidate variable, with the observed categories comprising NSIP, OP, NSIP+OP, DAD, and UIP+OP. However, the overall pattern distribution did not differ significantly between patients with and without an observed death in the training cohort (Fisher’s exact test, P = 0.603). In a previous related study, Zhang et al. reported prognostic information from total interstitial disease extent, diffuse distribution, and DAD pattern in patients with AE-IIM-ILD (10). Nevertheless, several visual-pattern categories in the present training cohort contained very few patients, and modeling the five-category variable would have required four indicator parameters despite only 20 observed deaths. We therefore did not construct a separate visual-pattern prognostic model. Consequently, the present study did not perform a formal head-to-head comparison with a conventional visual HRCT model and cannot establish that radiomics outperforms visual assessment or provides incremental prognostic value over it. Such a comparison should be undertaken in a larger independent cohort incorporating visual disease extent, distribution, and ILD pattern.

The final Rad-score was constructed as a linear combination of the two selected radiomics features: MF_RtLower_original_glszm_ZoneEntropy and RAW_RtUpper_original_shape_Maximum2DDiameterSlice. ZoneEntropy derived from the gray-level size zone matrix quantifies the randomness of the distribution of heterogeneous intensity zones, with higher values indicating greater image-texture heterogeneity. Maximum2DDiameterSlice is a shape feature describing the maximum two-dimensional diameter of the segmented region within the slice plane (23, 24, 39). Both features were retained through the multistep radiomics feature-selection procedure performed in the training cohort. Both selected radiomics signature were derived from the right lung. This may reflect cohort-, acquisition-, or feature-selection-specific effects and requires independent replication. However, neither feature reached conventional statistical significance in the two-feature radiomics CoxPH model, and their specific biological or histopathological correlates were not evaluated. Therefore, these features should be interpreted as quantitative imaging descriptors.

Performance varied across predictor strategies, algorithms, and evaluation metrics. CoxPH produced relatively similar training- and testing-cohort C-indices for the clinical and radiomics strategies, whereas the Combined-RSF configuration had the highest training-cohort C-index and was carried forward for exploratory analysis. Risk groups defined using the training-derived cutoff showed different observed survival trajectories in both cohorts. However, both model-configuration selection and cutoff derivation were based on the training cohort, and the small testing cohort and wide confidence intervals limit the precision of these findings. The study did not formally assess the incremental value of radiomics over clinical predictors or evaluate model-guided treatment. Thus, no conclusions regarding clinical utility can be drawn without validation in larger independent cohorts.

Several limitations must be acknowledged. First, this was a single-center retrospective study with a relatively small sample size (78 patients and 29 deaths). No formal *a priori* sample-size calculation was performed, and the held-out testing subset originated from the same center and contained only nine deaths. Although all feature-selection, hyperparameter-tuning, and model-development procedures were restricted to the training cohort, the limited sample size and relatively wide confidence intervals indicate that some performance optimism and model instability cannot be excluded. Second, the high-dimensional radiomics selection procedure used nominal significance thresholds during the initial screening stages without false discovery rate correction, and no formal ICC-based segmentation reproducibility analysis was performed. Although the same reconstruction-kernel category and slice thickness were used throughout the cohort, the use of a sharp bone kernel and different CT scanners and matrix sizes may limit the reproducibility of the selected radiomics features. Third, several visual ILD-pattern categories contained very few patients; consequently, no formal head-to-head comparison with a conventional visual HRCT prognostic model was performed. Finally, baseline pulmonary function tests (PFTs) were unavailable for many patients because of the severity of AE. ILD duration before AE admission could not be reliably determined, and standardized global myositis disease-activity measures were not routinely recorded at AE admission. Treatment decisions were retrospectively recorded rather than evaluated within a standardized model-guided treatment protocol. Future prospective studies with larger cohorts are needed to refine this prognostic model and evaluate its reproducibility and potential clinical utility.

In summary, this study developed and internally evaluated a machine learning-based prognostic model integrating clinical variables and a quantitative CT radiomics score for patients with AE-IIM-ILD. Among the 15 evaluated model configurations, the combined-RSF model incorporating PaO_2_/FiO_2_, arthritis-related symptoms, and Rad-score demonstrated the highest training-cohort C-index, effectively stratifying patients into high- and low-risk groups. These findings suggest that combining clinical and quantitative imaging information may provide complementary prognostic information. These exploratory findings require confirmation in larger independent multicenter cohorts before clinical application.

## Data Availability

The raw data supporting the conclusions of this article will be made available by the authors, without undue reservation.
