# Supplementary material for: Clinical variables and quantitative CT radiomics for mortality risk stratification after acute exacerbation of idiopathic inflammatory myopathy-associated interstitial lung disease
Source: Front Immunol. 2026 Oct 2;17:1917500. doi: 10.3389/fimmu.2026.1917500 (PMC13672662; doi:10.3389/fimmu.2026.1917500)
Supplement: Supplementary file 1 [file Supplementaryfile1.pdf]

Supplementary Material

1 Supplementary Method

1.1 Predictor strategies and model configurations

Three predictor strategies were evaluated. The clinical strategy included the PaO_2/FiO_2 ratio, NEU%, and arthritis-related symptoms. The radiomics strategy included the two final radiomics features, MF_RtLower_original_glszm_ZoneEntropy and RAW_RtUpper_original_shape_Maximum2DDiameterSlice, entered separately. The combined strategy included the PaO_2/FiO_2 ratio, arthritis-related symptoms, and the Rad-score, which was calculated as a linear combination of the two selected radiomics features weighted by their corresponding LASSO-Cox coefficients derived from the training cohort.

NEU% was not included in the combined strategy because multicollinearity assessment after integration of the Rad-score showed variance inflation factor values exceeding 5 for both NEU% and the PaO_2/FiO_2 ratio. NEU% also showed an attenuated association in the multivariable clinical CoxPH analysis ($P = 0.10$). The PaO_2/FiO_2 ratio was retained because of its stronger association with survival and its established clinical relevance, while NEU% was excluded to reduce redundancy and limit model complexity.

Five survival-learning algorithms were applied separately to each predictor strategy: Cox proportional hazards regression (CoxPH), Fast Kernel Survival Support Vector Machine (survival SVM), Gradient Boosting Survival Analysis (GBSA), Random Survival Forest (RSF), and Extra Survival Trees (EST). This factorial design resulted in 15 model configurations representing all combinations of the three predictor strategies and five algorithms. The radiomics and combined strategies were therefore treated as distinct approaches: the two radiomics features were entered separately in the radiomics models, whereas their composite Rad-score was entered as a single variable in the combined models.

1.2 Model training and hyperparameter optimization

All feature preprocessing, feature selection, model fitting, and hyperparameter optimization were conducted exclusively in the training cohort. The held-out testing cohort was not used during feature selection, preprocessing estimation, model fitting, hyperparameter tuning, or model-configuration selection.

Where feature scaling was required, preprocessing parameters were estimated from the training cohort and subsequently applied unchanged to the testing cohort. Hyperparameters for survival SVM, GBSA, RSF, and EST were optimized by grid search with five-fold cross-validation within the training cohort, using Harrell's concordance index as the optimization metric. CoxPH was fitted without algorithm-specific hyperparameter tuning. For each predictor strategy, the five algorithms were trained and evaluated independently; training- and testing-cohort performance estimates were also calculated separately.

Harrell's concordance index was defined as the primary measure of model discrimination. The configuration carried forward for subsequent exploratory risk-stratification and interpretation analyses

was identified according to its performance in the training cohort, with the training C-index used as the primary ranking criterion. Performance in the held-out testing cohort was not used to select the model configuration and was considered an internal evaluation of the training-derived model rather than external validation.

1.3 Performance estimation

Harrell's C-index was calculated separately in the training and held-out testing cohorts. Ninety-five percent confidence intervals were estimated using 1,000 nonparametric bootstrap resamples within each cohort.

Cumulative/dynamic time-dependent AUCs were calculated using inverse-probability-of-censoring weighting over the evaluable follow-up range of each cohort. The reported mean time-dependent AUC summarized discrimination across the displayed evaluable time points and was treated as a secondary descriptive measure. Because follow-up was administratively truncated at 365 days, the time-dependent AUC at the exact 365-day boundary was not estimated. Confidence intervals for the mean time-dependent AUC were obtained using bootstrap resampling.

The results of all 15 model configurations were reported without excluding configurations on the basis of poor or inconsistent performance. Accordingly, differences between the C-index and mean time-dependent AUC, including the metric-dependent behavior observed for some survival SVM configurations, were retained and interpreted descriptively.

1.4 Standalone analysis of the selected clinical variables

To characterize the unadjusted association and standalone discrimination of the three selected clinical variables, separate univariable CoxPH models were fitted for the PaO_2/FiO_2 ratio, NEU%, and arthritis-related symptoms in the training cohort. Regression coefficients, hazard ratios, 95% confidence intervals, exact P-values, and Harrell's C-indices were obtained for each variable.

The 180-day time-dependent AUC of each univariable CoxPH model was additionally calculated. Mean AUCs and 95% confidence intervals were estimated using 500 nonparametric bootstrap resamples. These standalone analyses were performed separately from the 15 multivariable model configurations and were not used for algorithm selection or construction of the combined predictor strategy.

1.5 Complementary CoxPH analyses

For transparency and inferential reporting, detailed CoxPH results were obtained for the clinical, radiomics, and combined predictor strategies. Regression coefficients, hazard ratios, 95% confidence intervals, z statistics, and exact P-values were reported for all predictors included in these CoxPH configurations. The combined CoxPH analysis was considered a complementary algorithm-specific analysis within the 15 evaluated configurations and should not be interpreted as the final combined model, because the model carried forward for subsequent exploratory analyses was selected independently according to the prespecified training-cohort discrimination criterion.

1.6 Exploratory risk stratification and model interpretation

For exploratory risk stratification, the risk-score cutoff was derived exclusively in the training cohort by maximizing the log-rank statistic while requiring at least five patients in each risk group. This training-derived cutoff was applied unchanged to the held-out testing cohort. Survival differences between the resulting high- and low-risk groups were evaluated using Kaplan–Meier curves and the log-rank test.

Shapley Additive Explanations were used to characterize the contributions of individual predictors to the predictions generated by the model carried forward for exploratory interpretation. SHAP values were interpreted as descriptions of model behavior and were not considered evidence of causal relationships or direct biological mechanisms.

2 Supplementary Figures and Tables

2.1 Figure S1

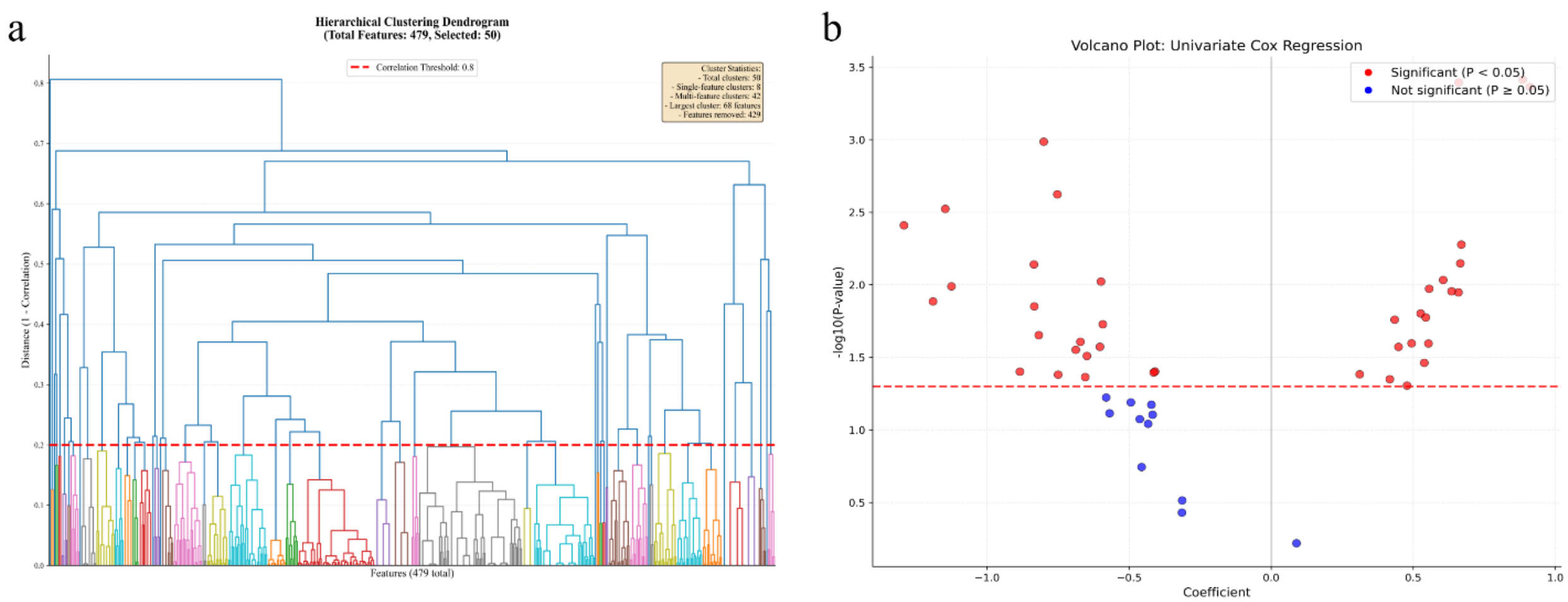

Supplementary Figure 1. Components of the radiomics feature selection procedure. (a) Hierarchical clustering used to reduce correlated features, with $|r| > 0.8$ indicating high correlation. (b) Volcano Plot of univariable Cox proportional hazards screening after preliminary filtering. The complete sequence and number of features retained at each stage are reported in Supplementary Table S4.

2.2 Figure S2

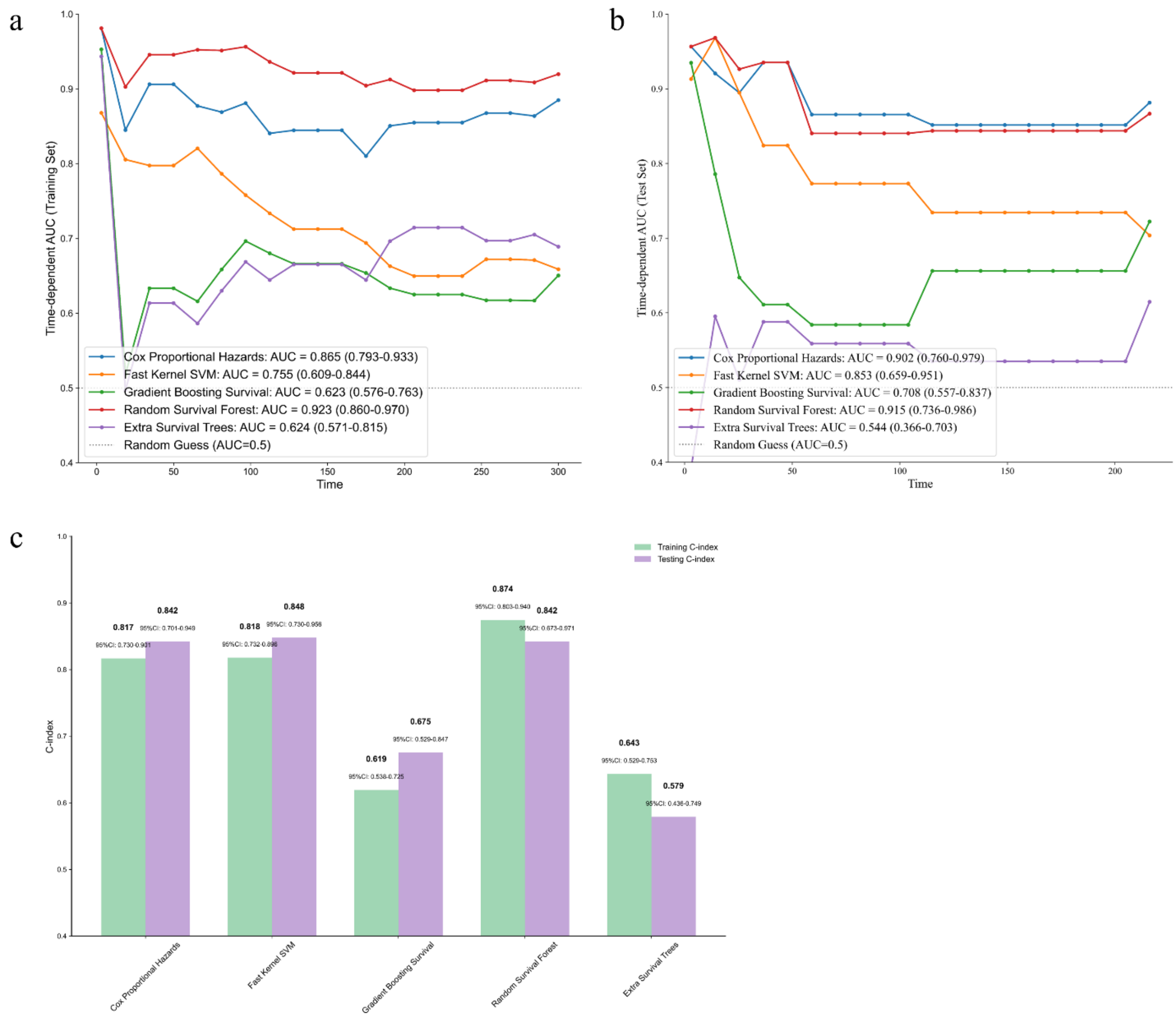

Supplementary Figure 2. Performance of five survival learning algorithms using the combined predictors. (a) Time-dependent AUCs in the training cohort. (b) Time-dependent AUCs in the held-out internal testing cohort. (c) Comparison of Harrell's concordance index (C-index) with 95% confidence intervals between the training and testing cohorts across the five models. The combined predictor strategy comprised PaO_2/FiO_2 ratio, arthritis-related symptoms, and Rad-score.

2.3 Figure S3

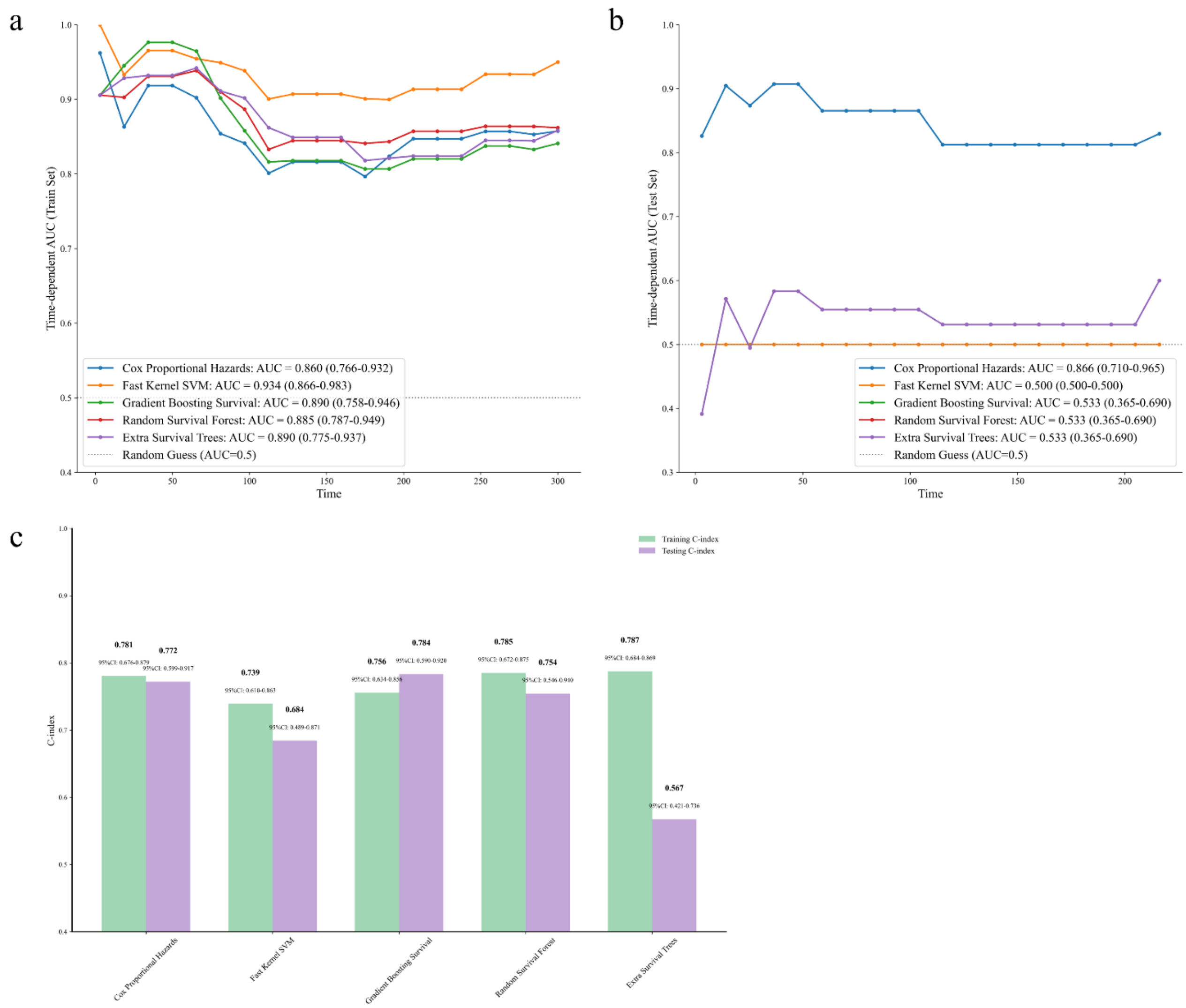

Supplementary Figure 3. Performance of five survival learning algorithms using the clinical predictors. (a) Time-dependent AUCs in the training cohort. (b) Time-dependent AUCs in the held-out internal testing cohort. (c) Comparison of Harrell’s concordance index (C-index) with 95% confidence intervals between the training and testing cohorts across the five models.

2.4 Figure S4

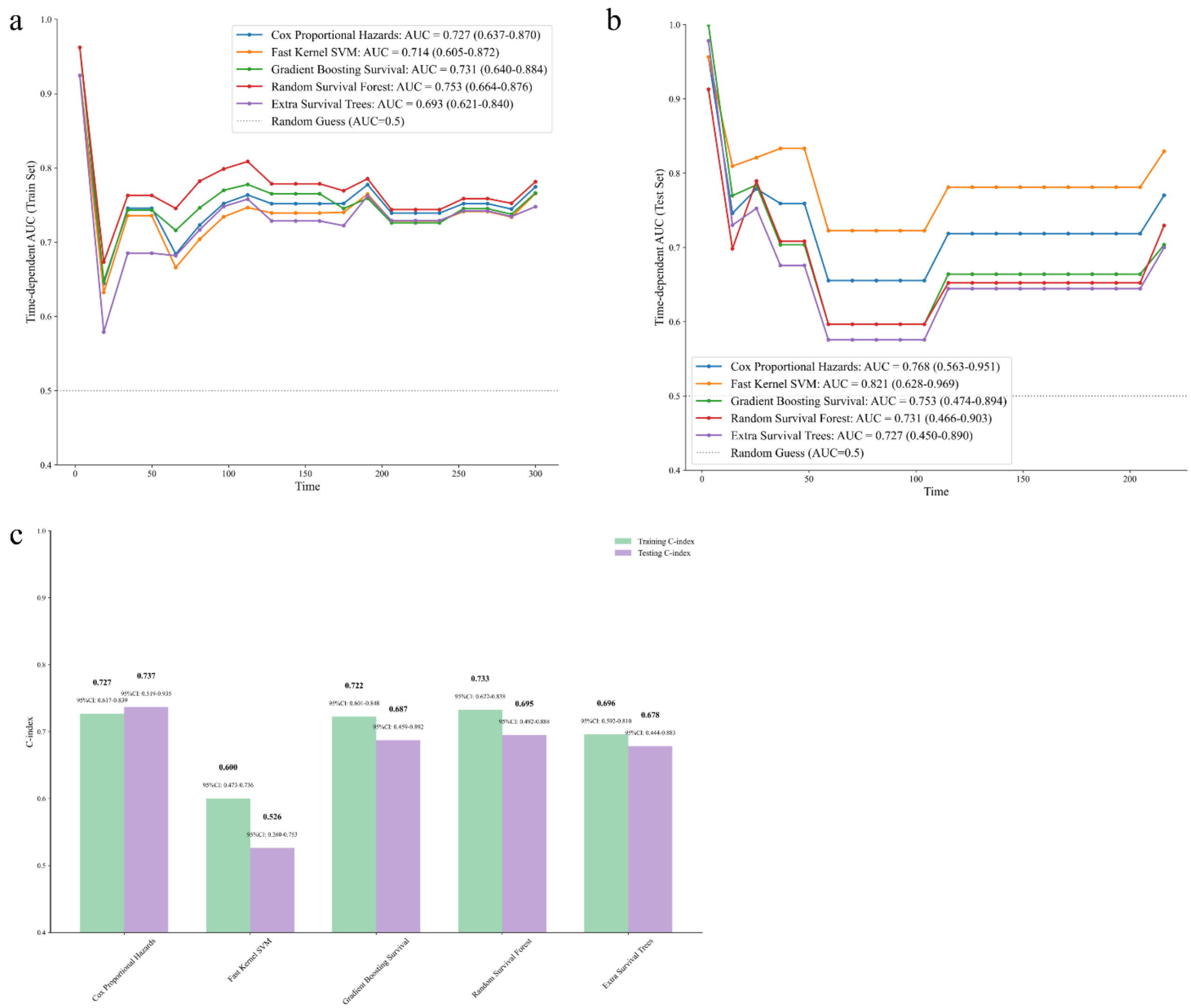

Supplementary Figure 4. Performance of five survival learning algorithms using the radiomics predictors. (a) Time-dependent AUCs in the training cohort. (b) Time-dependent AUCs in the held-out internal testing cohort. (c) Comparison of Harrell's concordance index (C-index) with 95% confidence intervals between the training and testing cohorts across the five models.

2.5 Table S1

Table S1 Comparison of clinical, laboratory, and radiologic characteristics at AE onset of the enrolled IIM-ILD patients in training and testing cohort (n = 78)

Characteristics§	Training Cohort (*n* = 54)		*P* value	Testing Cohort (*n* = 24)		*P* value
	No observed death (*n* = 34)	Death (*n* = 20)		No observed death (*n* = 15)	Death (*n* = 9)	
Gender (Female)	23(67.6)	15(75)	0.568	13(86.7)	6(66.7)	0.326#
Age, years	53.1(12.4)	53.6(8.8)	0.856	48.9(9.9)	53.3(9.2)	0.291
Temperature, ℃	36.5(0.5)	36.7(0.9)	0.349*	36.6(0.6)	36.9(0.6)	0.243*
Pulse, b/min	94(17.2)	100(17.8)	0.189	86(31.5)	102(34)	0.034
SBP, mmHg	115(15.3)	114(13.7)	0.774	110(26)	112(9)	0.835*
DBP, mmHg	74(9.2)	75(11)	0.643	73(11.3)	73(9.7)	0.892
BMI, kg/m^2	22(2.6)	20.5(4.1)	0.209	22.9(2.8)	19.9(2.7)	0.017
IIM disease duration, months	12.6(24.1)	6.5(10.8)	0.179*	7.5(13.6)	7.3(11)	0.474*
Smoking history						
Never/Ever	25(73.5)/9(26.5)	18(90)/2(10)	0.147	13(86.7)/2(13.3)	7(77.8)/2(22.2)	0.615#
IIM subtypes			0.410#			0.244#
DM	25(73.5)	12(60)	0.301	9(60)	7(77.8)	0.657#
PM	4(11.8)	3(15)	1.00#	2(13.3)	1(11.1)	1.00#
ASyS	3(8.8)	1(5)	1.00#	4(26.7)	0(0)	0.259#
OM	2(5.9)	4(20)	0.179#	0(0)	1(11.1)	0.375#
Symptoms						
Fever	6(17.6)	6(30)	0.326#	3(20)	4(44.4)	0.356#
Cough/Expectoration	20(58.8)	10(50)	0.529	9(60)	3(33.3)	0.40#
Dyspnea	13(38.2)	9(45)	0.625	11(73.3)	4(44.4)	0.212#
Cutaneous manifestations	16(47.1)	10(50)	0.835	6(40)	4(44.4)	1.00#
Myalgia/Myasthenia	18(52.9)	14(70)	0.218	9(60)	6(66.7)	1.00#

Arthralgia/Joint swelling	6(17.6)	10(50)	0.012	2(13.3)	3(33.3)	0.326#
Comorbidities						
Hypertension	6(17.6)	8(40)	0.07	1(6.7)	1(11.1)	1.00#
Diabetes mellitus	7(20.6)	6(30)	0.517#	1(6.7)	2(22.2)	0.533#
Coronary heart disease	5(14.7)	6(30)	0.294#	0(0)	1(11.1)	0.375#
Other disease	20(58.8)	12(60)	0.932	4(26.7)	7(77.8)	0.033#
No-Comorbidities	11(32.4)	4(20)	0.328	10(66.7)	2(22.2)	0.089#
MSA/MAA profile						
Anti-MDA5 Ab	6(17.6)	4(20)	1.00#	2(13.3)	2(22.2)	0.615#
Anti-ARS Ab	13(38.2)	3(15)	0.071	8(53.3)	1(11.1)	0.08#
Anti-Mi-2 Ab	1(2.9)	0(0)	1.00#	1(6.7)	0(0)	1.00#
Anti-SRP Ab	2(5.9)	0(0)	0.525#	-	-	-
MSA Negative	6(17.6)	2(10)	0.695#	1(6.7)	3(33.3)	0.13#
Anti-Ro-52 Ab	20(58.8)	12(60)	0.932	11(73.3)	5(55.6)	0.412#
MSA/MAA unavailable	6(17.6)	11(55)	0.004	3(20)	3(33.3)	0.635#
Laboratory						
CK, U/L	72.5(1100)	67.5(163)	0.548*	76(188)	182(346)	0.952*
CK-MB, U/L	16(22.9)	19.4(10.8)	0.893*	17(16.5)	17.2(11.3)	0.858*
LDH, U/L	361(276)	403(246)	0.255*	376(544)	623(171)	0.138*
HBDH, U/L	301(164)	340(110)	0.139*	229(389)	469(206)	0.123*
ALT, U/L	31.5(43)	38.5(50)	0.768*	27(76.2)	63(75.5)	0.551*
AST, U/L	41(48.3)	34(52)	0.713*	49.7(67.2)	85(96.5)	0.233*
WBC, 10^9/L	5.9(4.6)	7.6(3.6)	0.099*	8.3(3.8)	8.7(3.2)	0.817
Platelet, 10^9/L	206(101)	208(113)	0.747*	247(122)	215(70.3)	0.479
Hemoglobin, g/L	128(20)	116(18.3)	0.014*	126(12.8)	120(20.9)	0.422
Neutrophil, 10^9/L	4.6(3.6)	6.2(3.6)	0.025*	6.1(2.6)	7.4(4.0)	0.482*

Neutrophil %	77.8(19.5)	87.8(7.1)	<0.001	80.1(8.5)	83(6.4)	0.395
Lymphocyte, 10^9/L	0.83(0.71)	0.56(0.56)	0.068*	0.84(1.1)	0.84(0.2)	0.929*
Lymphocyte%	12.9(15.7)	7.7(6.5)	0.004*	10.2(6.9)	9.5(5.3)	0.411*
CRP, mg/L	10(43.7)	48.1(66.3)	0.038*	10(28.7)	12.1(41)	0.261*
ESR, mm/h	32(42)	43.5(43.3)	0.121*	23(18.5)	37(19)	0.21*
Ferritin, ng/ml	523(775)	309(772)	0.445*	278(696)	979(2174)	0.283*
pro-BNP, ng/ml	237(473)	403(843)	0.109*	134(327)	249(156)	0.835*
PaO_2/FiO_2, mmHg	263(91.9)	183(85.4)	0.003	254(83.5)	159(86.9)	0.014
PCT, ng/ml	0.05(0.01)	0.11(0.34)	0.025*	0.05(0.12)	0.08(0.13)	0.352*
ILD pattern			0.603#			
NSIP	9(26.5)	5(25)	0.905	2(13.3)	1(11.1)	1.00#
OP	9(26.5)	5(25)	0.905	4(26.7)	2(22.5)	1.00#
NSIP+OP	10(29.4)	5(25)	0.727	7(46.7)	3(33.3)	0.678#
DAD	2(5.9)	4(20)	0.179#	2(13.3)	2(22.2)	0.615#
UIP+OP	4(11.8)	1(5)	0.64#	0(0)	1(11.1)	0.375#
Treatment after AE						
CS pulse therapy	4(11.8)	3(15)	1.00#	1(6.7)	1(11.1)	1.00#
Cyclophosphamide	8(23.5)	2(10)	0.291#	3(20)	0(0)	0.266#
Cyclosporine	3(8.8)	1(5)	1.00#	1(6.7)	0(0)	1.00#
Mycophenolate mofetil	8(23.5)	1(5)	0.131#	3(20)	1(11.1)	1.00#
hydroxychloroquine	11(32.4)	4(20)	0.328	3(20)	0(0)	0.266#
Thalidomide	5(14.7)	1(5)	0.395#	2(13.3)	1(11.1)	1.00#
Tofacitinib	3(8.8)	1(5)	1.00#	3(20)	0(0)	0.266#
Tocilizumab	2(5.9)	1(5)	1.00#	-	-	-
Tacrolimus	1(2.9)	0(0)	1.00#	2(13.3)	0(0)	0.511#
IVIG	5(14.7)	8(40)	0.05#	0(0)	3(33.3)	0.042#
Antifibrotic therapy	3(8.8)	1(5)	1.00#	1(6.7)	1(11.1)	1.00#

Antimicrobial therapy	3(8.8)	1(5)	1.00#	10(66.7)	9(100)	0.118#
Supplemental oxygen therapy	25(73.5)	19(95)	0.072#	10(66.7)	8(88.9)	0.351#
Mechanical ventilation	3(8.8)	4(20)	0.403#	1(6.7)	5(55.6)	0.015#

*§Data are presented as mean (SD) or median [IQR] for continuous variables as appropriate, and number (frequency) (%) for categorical variables. * Mann-whitney U test; # Fisher's exact test.*

Abbreviations: AE, acute exacerbation; IIM, idiopathic inflammatory myopathies; ILD, interstitial lung disease; SBP, systolic blood pressure; DBP, diastolic blood pressure; BMI, body mass index; DM, dermatomyositis; PM, polymyositis; ASyS, anti-synthetase syndrome; OM, overlap myositis; MAAs, myositis-associated autoantibodies; MSAs, myositis specific autoantibodies; MDA5, melanoma differentiation-associated protein 5; Ab, antibody; ARS, anti-aminoacyl-tRNA synthetase antibodies; CK, creatine kinase; CK-MB, creatine kinase-MB isoenzyme; LDH, lactate dehydrogenase; HDBH, hydroxybutyrate dehydrogenase; ALT, alanine transaminase; AST, aspartate transaminase; WBC, white blood cell; CRP, c-reactive protein; ESR, erythrocyte sedimentation rate; pro-BNP, pro-brain natriuretic peptide; PaO_2/FiO_2, arterial oxygen partial pressure/fraction of inspired oxygen; PCT, procalcitonin; NSIP, nonspecific interstitial pneumonia; OP, organizing pneumonia; DAD, diffuse alveolar damage; UIP, usual interstitial pneumonia. CS, corticosteroids; IVIG, intravenous immunoglobulin.

Radiological Pattern Notes: ILD patterns were evaluated at AE onset according to the 2013 ATS/ERS multidisciplinary consensus statement. 1. NSIP, nonspecific interstitial pneumonia; OP, organizing pneumonia; DAD, diffuse alveolar damage. 2. UIP+OP indicates chronic usual interstitial pneumonia (UIP) features superimposed with acute organizing pneumonia (OP) infiltrates at AE presentation.

ILD duration before AE admission could not be reliably determined from the retrospective records.

Cutaneous manifestations recorded at AE admission included Gottron's papules/sign, heliotrope rash, shawl sign, V-sign, mechanic's hands, Raynaud phenomenon, and skin ulceration.

2.6 Table S2

Table S2. Stepwise selection of clinical predictors and definition of the clinical and combined predictor strategies in the training cohort

	Variables retained or evaluated	N	Criterion or reason
Initial comparison	All clinical, laboratory, treatment-related, and visual HRCT variables reported in Supplementary Table S1	68	Between-group comparisons were performed in the training cohort. Variables describing treatments administered after AE onset were reported descriptively but were not eligible for baseline prognostic modeling.
Between-group screening	Arthritis-related symptoms; MSA/MAA testing unavailable ("Miss"); HGB; NEU; NEU%; LYMPH%; CRP; PaO_2/FiO_2 ratio; PCT	9	$P < 0.05$
Removal of the nonbiological variable	Arthritis-related symptoms; HGB; NEU; NEU%; LYMPH%; CRP; PaO_2/FiO_2 ratio; PCT	8	"Miss" was excluded because it represented the availability of serological testing rather than a biological patient characteristic.
Univariable CoxPH screening	PaO_2/FiO_2 ratio; NEU%; arthritis-related symptoms; LYMPH%; CRP	5	Univariable CoxPH $P < 0.05$
Cross-validated importance ranking	PaO_2/FiO_2 ratio; NEU%; arthritis-related symptoms	3	The three highest-ranked variables in five-fold cross-validated CoxPH and RSF feature-importance analyses

All clinical feature-selection procedures were performed exclusively in the training cohort.

2.7 Table S3

Table S3. Multicollinearity assessment of variables initially considered for the combined predictor strategy

	VIF	Tolerance
PaO_2/FiO_2 ratio	5.24	0.191
NEU%	5.38	0.186
Arthritis-related symptoms	1.53	0.654
Rad-score	1.12	0.892

2.8 Table S4

Table S4. Radiomic feature reduction results

Processing / Filtering	Inclusion / Exclusion Criteria	Retained Features (N)
Image Pre-processing	Fixed bin width (25) & Wavelet decomposition	3232
Low-Variance Filtering	Removed features with near-zero variance (variance < 0.01)	2223
Between-Group Comparison	Retained features with P < 0.05 in training set	479
Hierarchical Clustering	Removed collinear features with |r| > 0.8	50
Univariate Cox Screening	Retained features with univariate P < 0.05	39
LASSO-Cox Penalization	5-fold cross-validated L1 regularization	6
CV Importance Ranking	Top-ranked dominant features in 5-fold CV Cox and RSF	2

2.9 Table S5

Table S5. Standalone prognostic performance of the three selected clinical variables in the training cohort

	β	HR (95% CI)	P value	Harrell's C-index	Bootstrap mean 180-day time-dependent AUC (95% CI)
PaO_2/FiO_2 ratio	−0.0092	0.99 (0.99–1.00)	0.002	0.71	0.842 (0.646–0.988)
NEU%	0.0830	1.09 (1.03–1.14)	0.002	0.73	0.555 (0.289–0.805)
Arthritis-related symptoms	1.1009	3.01 (1.24–7.27)	0.015	0.62	0.542 (0.361–0.750)

2.10 Table S6

Table S6. Predictive performance metrics of five machine learning survival algorithms using three predictor strategies in training and testing cohorts.

Predictor strategy	Algorithm	Training C-index (95% CI)	Testing C-index (95% CI)	Training mean time-dependent AUC (95% CI)	Testing mean time-dependent AUC (95% CI)
combined	CoxPH	0.817 (0.730–0.901)	0.842 (0.701–0.949)	0.865 (0.793–0.933)	0.902 (0.760–0.979)
	survival SVM	0.818 (0.732–0.896)	0.848 (0.730–0.956)	0.755 (0.609–0.844)	0.853 (0.659–0.951)
	GBSA	0.619 (0.538–0.725)	0.675 (0.529–0.847)	0.623 (0.576–0.763)	0.708 (0.557–0.837)
	**RSF**	**0.874 (0.803–0.940)**	0.842 (0.673–0.971)	**0.923 (0.860–0.970)**	**0.915 (0.736–0.986)**
	EST	0.643 (0.529–0.753)	0.579 (0.436–0.749)	0.624 (0.571–0.815)	0.544 (0.366–0.703)
clinical	CoxPH	0.781 (0.676–0.879)	0.772 (0.599–0.917)	0.860 (0.766–0.932)	0.866 (0.710–0.965)
	survival SVM	0.739 (0.610–0.863)	0.684 (0.489–0.871)	0.934 (0.866–0.983)	0.500 (0.500–0.500)
	GBSA	0.756 (0.634–0.856)	0.784 (0.590–0.920)	0.890 (0.758–0.946)	0.533 (0.365–0.690)

	RSF	0.785 (0.672–0.875)	0.754 (0.546–0.940)	0.885 (0.787–0.949)	0.533 (0.365–0.690)
	EST	0.787 (0.684–0.869)	0.567 (0.421–0.736)	0.890 (0.775–0.937)	0.533 (0.365–0.690)
radiomics	CoxPH	0.727 (0.617–0.839)	0.737 (0.519–0.935)	0.727 (0.637–0.870)	0.768 (0.563–0.951)
	survival SVM	0.600 (0.473–0.736)	0.526 (0.260–0.753)	0.714 (0.605–0.872)	0.821 (0.628–0.969)
	GBSA	0.722 (0.601–0.848)	0.687 (0.459–0.892)	0.731 (0.640–0.884)	0.753 (0.474–0.894)
	RSF	0.733 (0.622–0.838)	0.695 (0.492–0.888)	0.753 (0.664–0.876)	0.731 (0.466–0.903)
	EST	0.696 (0.592–0.810)	0.678 (0.444–0.883)	0.693 (0.621–0.840)	0.727 (0.450–0.890)

2.11 Table S7

Table S7. Detailed Cox proportional hazards regression results for the clinical, radiomics, and combined predictor strategies in the training cohort

Predictor strategy	Predictor	β	HR (95% CI)	z statistic	P value
clinical	PaO_2/FiO_2 ratio	-0.01	0.99 (0.98–1.00)	-2.89	0.004
	Arthritis-related symptoms	1.33	3.79 (1.42–10.08)	2.67	0.008
	NEU%	0.05	1.05 (0.99–1.11)	1.63	0.104
radiomics	MF_RtLower_original_glszm_ZoneEntropy	0.34	1.41 (0.92–2.15)	1.59	0.111
	RAW_RtUpper_original_shape_Maximum2DDiameterSlice	-0.46	0.63 (0.38-1.04)	-1.80	0.072
combined	PaO_2/FiO_2 ratio	-0.01	0.99 (0.98–1.00)	-2.89	0.004
	Arthritis-related symptoms	1.27	3.54 (1.28–9.79)	2.44	0.015
	Rad-score	0.88	2.42 (1.03–5.69)	2.02	0.044
